# Late Relapse After Autologous Hematopoietic Stem Cell Transplantation in AQP4-IgG–Positive NMOSD

**DOI:** 10.1001/jamanetworkopen.2025.5989

**Published:** 2025-04-21

**Authors:** Nisa Vorasoot, Kyle M. Blackburn, Linda Nguyen, Melissa L. Bush, Sarah M. Jenkins, Jiraporn Jitprapaikulsan, James P. Fryer, Sarosh R. Irani, Eoin P. Flanagan, Sean J. Pittock

**Affiliations:** 1 Department of Neurology, Mayo Clinic, Rochester, Minnesota; 2 Department of Laboratory Medicine and Pathology, Mayo Clinic, Rochester, Minnesota; 3 Center for MS and Autoimmune Neurology, Mayo Clinic, Rochester, Minnesota; 4 Division of Neurology, Department of Medicine, Faculty of Medicine, Khon Kaen University, Khon Kaen, Thailand; 5 Department of Neurology, UT Southwestern Medical Center, Dallas, Texas; 6 Department of Quantitative Health Sciences, Division of Clinical Trials and Biostatistics, Mayo Clinic, Rochester, Minnesota; 7 Division of Neurology, Department of Medicine, Faculty of Medicine, Siriraj Hospital, Mahidol University, Bangkok, Thailand; 8 Departments of Neurology and Neurosciences, Mayo Clinic, Jacksonville, Florida

## Abstract

This case series examines long-term outcomes for 2 aquaporin-4-IgG (AQP4-IgG)–positive patients with neuromyelitis optica spectrum disorder (NMOSD) who underwent autologous hematopoietic stem cell transplantation with a nonmyeloablative conditioning regimen and rituximab.

## Introduction

Neuromyelitis optica spectrum disorder (NMOSD) is an autoimmune disease driven by pathogenic antibodies targeting aquaporin-4 (AQP4), leading to relapsing optic neuritis and myelitis. Without treatment, up to one-half of patients may develop severe disability, including blindness or paralysis, within 5 years.^1^ Acute NMOSD attacks are managed with high-dose corticosteroids and plasmapheresis, while long-term preventive treatments include rituximab, eculizumab, satralizumab, inebilizumab, and ravulizumab, all of which reduce relapse rates.^1^ At the time, when no FDA-approved medications were available for NMOSD, autologous hematopoietic stem cell transplantation (AHSCT) was explored as a treatment option for refractory cases, following its success in treating severe multiple sclerosis (MS).^2,3^ In 2019, Burt et al^4^ reported AQP4-IgG seroreversion and up to 5 years of remission in patients with NMOSD post-AHSCT.^4^ However, while AHSCT may induce remission, long-term outcomes beyond 5 years remain underreported.

## Methods

In this case series, we reviewed 2 AQP4-IgG–positive patients with NMOSD who underwent AHSCT with a nonmyeloablative conditioning regimen and rituximab as part of an open-label cohort study (2008-2016).^4^ Both patients achieved AQP4-IgG seronegativity (seroreversion) and remained attack-free for at least 5 years before relapsing more than 10 years later. Serum samples were retested for AQP4-IgG status and complement activation using flow cytometry on live AQP4-transfected cells, following previously established protocols.^4^ This study was approved by the Mayo Clinic institutional review board, and written informed consent was obtained from both patients. This review followed the reporting guideline for case series. Statistical analysis was performed in April 2024 using R version 4.1.1 (R Project for Statistical Computing).

## Results

**Figure 1.**
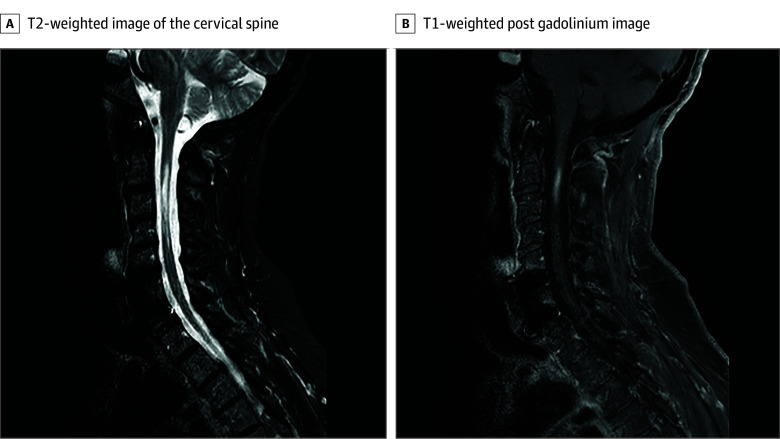
Magnetic Resonance Imaging (MRI) of Active Myelitis in Cervical Spine The MRI images show active myelitis at the C2 to C5 levels on the T2-weighted image of the cervical spine (A) and the T1-weighted post gadolinium image (B).

A woman in her 60s, diagnosed with NMOSD in her 30s in 2002, experienced multiple episodes of optic neuritis and myelitis despite treatment with azathioprine and mycophenolate. AQP4-IgG was detected in 2005 (titer 1:100). She underwent AHSCT in 2013 after continued disease activity. By 2017, she achieved seronegativity and remained relapse-free until January 2023, a decade post-AHSCT (6 years post-seroreversion), when she presented with myelitis confirmed by magnetic resonance imaging (MRI), showing active longitudinal extensive transverse myelitis (LETM) at the C2 to C5 levels of the cervical spine (Figure 1) and seroconverted to positive (AQP4-IgG titer 1:100 000). She was treated with intravenous methylprednisolone (IVMP), plasma exchange, and started on satralizumab, but unfortunately developed fatal pulmonary aspergillosis in June 2023.

A woman in her 70s, diagnosed with NMOSD in her 60s in 2010 after recurrent LETM, experienced multiple relapses despite treatment with corticosteroids and azathioprine. AQP4-IgG seropositivity (titer 1:100) was confirmed, and she underwent AHSCT in November 2010. She remained relapse-free and seronegative until March 2022, when she relapsed with area postrema syndrome (AQP4-IgG titer 1:1000). She was treated with corticosteroids and transitioned to eculizumab for relapse prevention.

**Figure 2.**
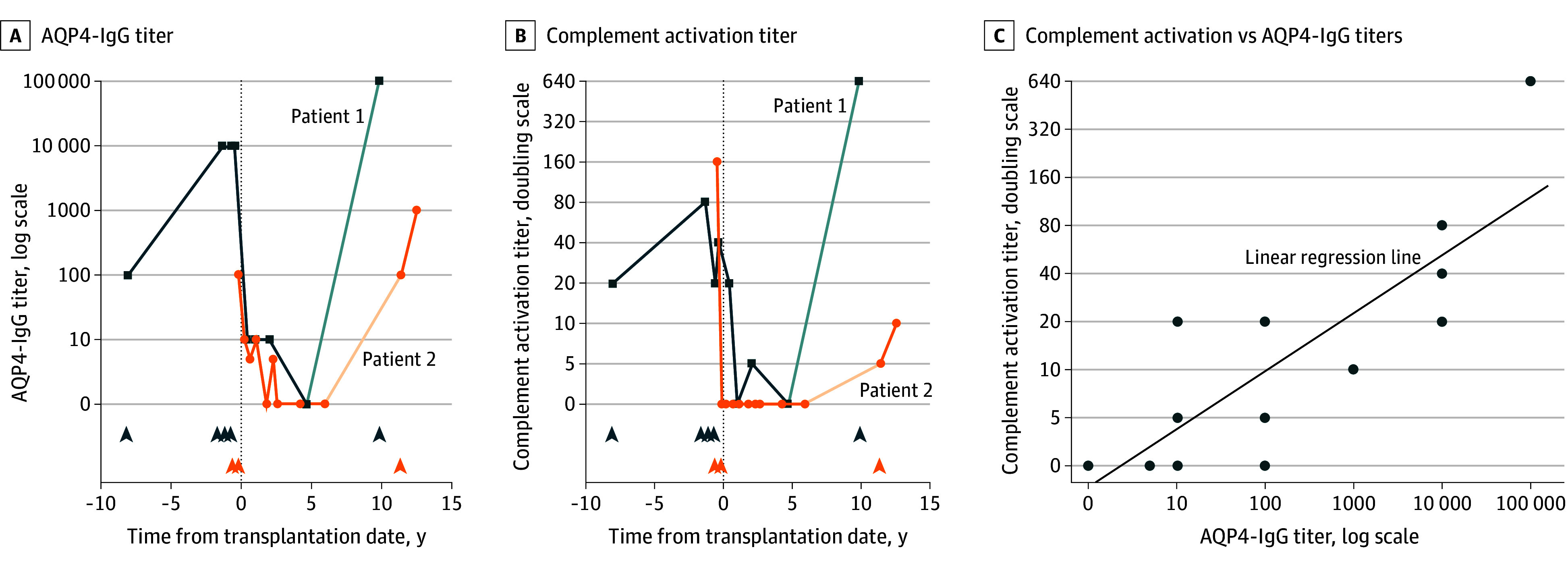
Aquaporin-4-IgG (AQP4-IgG) and Complement Activation Titers for Patients 1 and 2 The AQP4-IgG titers (A) demonstrate peaks during attacks in both patients, followed by seronegativity after autologous hematopoietic stem cell transplantation (AHSCT). However, 10 years post-AHSCT, both patients experienced seroconversion and subsequent relapses. Notably, during relapses post-AHSCT, AQP4-IgG titers were higher than before AHSCT in both cases (patient 1: pre-AHSCT titer 1:10 000; post-AHSCT titer 1:100 000; patient 2: pre-AHSCT titer 1:100; post-AHSCT titer 1:1000). The AQP4-IgG complement activation titers (B) exhibit elevation during attacks in both patients, subsequently decreasing after AHSCT, but increasing again during relapse 10 years post-AHSCT. The scatter plot (C) shows a correlation of 0.86 between AQP4-IgG serostatus and complement activation. Patient 1 is represented in blue, patient 2 in orange, and the lighter shades of blue and orange indicate intervals without follow-up; arrowheads indicate clinical attacks.

In both patients, AQP4-IgG titers peaked during relapses and returned to seronegativity following AHSCT. However, 10 years after AHSCT, both experienced seroconversion to AQP4-IgG positivity and relapsed. Notably, AQP4-IgG titers during relapses exceeded pre-AHSCT levels, correlating with complement activation, suggesting a pathogenic role for AQP4-IgG in driving disease activity (Figure 2).

## Discussion

In this study, we provided unique long-term follow-up data, documenting AQP4-IgG seroconversion and relapses more than 10 years after AHSCT in 2 patients with NMOSD. These findings extended earlier work of Burt et al,^4^ which demonstrated that 80% of patients with NMOSD remained relapse-free for up to 5 years post-AHSCT. Long-term remission post-AHSCT likely results from immune reprogramming and B-cell depletion.^3^ However, our findings suggest that AQP4-reactive B cells may eventually repopulate, leading to seroconversion and recurrence.

The reemergence of AQP4-reactive B cells, gradually entering the memory B-cell compartment and producing high-affinity AQP4-IgG antibodies, may explain these late relapses.^5,6^ Although AHSCT offers extended relapse-free periods, it may not eliminate the risk of disease reactivation entirely. Long-term monitoring of AQP4-IgG titers post-AHSCT is crucial for detecting early signs of recurrence. Based on our findings, we recommend testing AQP4-IgG titers every 6 months in patients who have undergone AHSCT. If seroconversion occurs, we suggest reinitiating therapy with FDA-approved medications based on the patient’s clinical history and relapse risk. A limitation of this study is that it included only 2 patients from a previous cohort study.

Our findings highlight the need for long-term clinical and serological monitoring and question the durability of AHSCT in achieving permanent remission. Larger, long-term studies are needed to assess its efficacy against alternative therapies.

## Notes

Supplement.
Data Sharing Statement
