# Supplementary material for: Late Relapse After Autologous Hematopoietic Stem Cell Transplantation in AQP4-IgG–Positive NMOSD
Source: JAMA Netw Open. 2025 Apr 21;8(4):e255989. doi: 10.1001/jamanetworkopen.2025.5989 (PMC12013349; doi:10.1001/jamanetworkopen.2025.5989)
Supplement: Supplement. — Data Sharing Statement [file jamanetwopen-e255989-s001.pdf]

Data Sharing Statement

Vorasoot. Late Relapse After Autologous Hematopoietic Stem Cell Transplantation in AQP4-IgG–Positive NMOSD. *JAMA Netw Open*. Published April 21, 2025.
doi:10.1001/jamanetworkopen.2025.5989

Data
Data available: No

Additional Information
Explanation for why data not available: All data is already provided in the manuscript.
